# A functional antagonism between RhoJ and Cdc42 regulates fibronectin remodelling during angiogenesis

**DOI:** 10.1080/21541248.2020.1809927

**Published:** 2020-08-28

**Authors:** Ananthalakshmy Sundararaman, Harry Mellor

**Affiliations:** a Cardiovascular Diseases and Diabetes Biology, Rajiv Gandhi Centre for Biotechnology (RGCB), Thiruvananthapuram, Kerala, India; b School of Biochemistry, Biomedical Sciences Building, University of Bristol, Bristol, UK

## Abstract

Angiogenesis is the formation of new blood vessels from pre-existing ones. Angiogenesis requires endothelial cells to change shape and polarity, as well as acquire the ability to directionally migrate ‒ processes that are classically regulated by the Rho family of GTPases. RhoJ (previously TCL) is an endothelium enriched Rho GTPase with a 78% amino acid similarity to the ubiquitously expressed Cdc42. In our recent publication, we demonstrate that α5β1 integrin co-traffics with RhoJ. RhoJ specifically represses the internalization of the active α5β1 conformer, leading to a reduced ability of endothelial cells to form fibronectin fibrils. Surprisingly, this function of RhoJ is in opposition to the role of Cdc42, a known driver of fibrillogenesis. Intriguingly, we discovered that the competition for limiting amounts of the shared effector, PAK3, could explain the ability of these two Rho GTPases to regulate fibrillogenesis in opposing directions. Consequently, RhoJ null mice show excessive fibronectin deposition around retinal vessels, possibly due to the unopposed action of Cdc42. Our work suggests that the functional antagonism between RhoJ and Cdc42 could restrict fibronectin remodelling to sites of active angiogenesis to form a provisional matrix for vessel growth. One correlate of our findings is that RhoJ dependent repression of fibronectin remodelling could be atheroprotective in quiescent vessels.

Rho GTPases are central regulators of the cytoskeleton [1,5]. Rho GTPases were discovered quite serendipitously as being small molecular weight proteins homologous to the ras family of GTPases (Ras homologous-Rho) [6]. We now know that this family comprises of 21 proteins in humans [7] of which RhoA, Rac1 and Cdc42 are the archetypical members. The entire Rho family of GTPases has arisen from a single ancestral gene through several gene duplication events generating many in-and-out-paralogs [8]. Generally, duplicated genes are unstable and are lost from the genome unless there is a selective pressure on them to evolve new functions (neofunctionalisation) or partition old functions (subfunctionalisation) [9]. Of specific interest to us is the relationship between RhoJ and the ancestral Cdc42 genes. Two rounds of whole-genome duplication in the ancestral vertebrate [9] is believed to have resulted in ancestral Cdc42 gene duplicating to give rise to the present Cdc42, RhoQ, and RhoJ in humans. The emergence of the adaptive immune system, a closed blood vascular system, and more complex neural systems in the vertebrates could have led to neofunctionalisation and fixation of these newer RhoGTPases. Previous studies have indicated that Cdc42 and RhoJ could have several shared functions, owing to shared effector binding [2,3]. Notably, while endothelium specific Cdc42 deletion in mice leads to severe defects in lumen formation during embryonic development, causing lethality at E9-10 [10], RhoJ null mice have a milder phenotype with reduced number and branching of large vessels [11]. These results suggested that RhoJ could have distinct roles compared to Cdc42 in angiogenesis.

To study the unique roles of RhoJ in endothelial cells, we exploited the difference in subcellular localization of RhoJ and Cdc42. RhoJ is enriched in both small and large vesicles located both at the cell periphery and in the perinuclear region, while Cdc42 has a more homogenous distribution with enrichment at the Golgi. Therefore, we hypothesized that RhoJ might have roles in the trafficking of endothelial cargo not shared with Cdc42. To identify these, we used density gradient ultracentrifugation to enrich for RhoJ^+^ vesicles, separating out any RhoJ that is plasma membrane bound [4]. Further, we sorted out RhoJ^+^ vesicles with a particle sorter and identified proteins present in the same vesicle as RhoJ by mass spectrometry. We followed up on the highest hit, the integrin α5β1 and found that RhoJ did not affect the total receptor levels or its distribution. Surprisingly, RhoJ had a conformation-specific effect on α5β1 integrin and siRNA mediated RhoJ depletion increased steady-state levels of active α5β1. To further understand the fate of the active receptor upon RhoJ modulation, we undertook receptor internalization and recycling assays wherein the surface receptors were biotinylated and chased across multiple time points. RhoJ had a clear effect on the internalization of active α5β1. Lack of RhoJ doubled the internalization rates suggesting that RhoJ acts as a brake on active receptor internalization. RhoJ also diverted the active α5β1 integrin to LAMP1^+^ compartments poised for degradation while reducing their recycling through the TGN46^+^ compartments (Figure 1).

**Figure 1.**
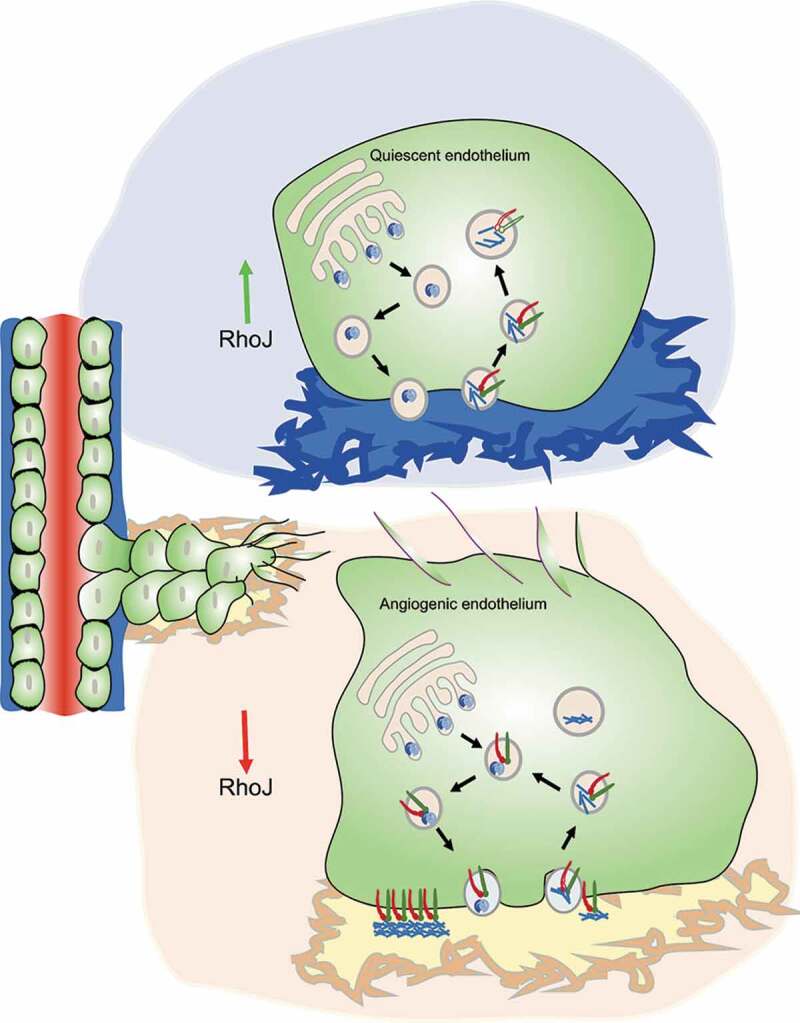
Quiescent endothelial cells rest on a laminin rich basement membrane matrix [38] (blue). High RhoJ activity in these cells prevents fibronectin bundling and deposition as fibrils. RhoJ inhibits the uptake of ligand-bound α5β1 and diverts the receptors into a degradative fate. At the onset of angiogenic sprouting, a fibronectin rich provisional matrix is laid out (yellow). RhoJ inactivation by angiogenic growth factors promotes active α5β1 internalization and trafficking through the post-Golgi compartments to promote fibronectin fibrillogenesis

Active α5β1 integrin trafficking is linked to the ability of cells to remodel fibronectin [12]. Since we saw that RhoJ reduced the flux of active integrin α5β1 in the cells, we hypothesized that RhoJ would negatively regulate fibronectin remodelling. Using microscopy and biochemical deoxycholate insolubility assays, we showed that RhoJ indeed inhibited the bundling of cell-derived fibronectin (EDA-FN) into fibrils. Since the secretion of fibronectin is not perturbed upon RhoJ modulation, we think that the polarized and targeted release of fibronectin at basal fibrillar adhesions, which requires the coupling of fibronectin secretion with integrin trafficking [13], is perturbed resulting in low fibril formation when RhoJ is active.

Since Cdc42 has been reported to promote fibrillogenesis in fibroblasts [14], we enquired if this was true for endothelial cells as well. Our results suggested that indeed Cdc42 was a positive regulator of fibrillogenesis in endothelial cells. This led to the intriguing question as to how RhoJ and Cdc42 drive cell behaviour in opposing directions. After failing to detect unique high-affinity interactors of RhoJ that did not bind Cdc42, we explored a common effector PAK3 that binds to both the Rho GTPases. PAK3 was also found to be a positive regulator of fibrillogenesis. However, upon co-expression of active RhoJ, PAK3 could no longer drive fibrillogenesis. This suggested that the RhoJ-PAK3 complex inhibited fibrillogenesis while the Cdc42-PAK3 complex activated the same. Our results indicated that RhoJ and Cdc42 might compete for limiting amounts of PAK3 to regulate fibrillogenesis. Indeed, a previous study showed that PAK3 is limiting in cells and is competed for by Nck, βPIX and paxillin α [15]. PAK3 is known to heterodimerise with PAK1 [16], and our data revealed that PAK1 also regulates fibrillogenesis similar to PAK3.

Fibronectin deposition with the EIIIA domain (EDA-FN) happens almost exclusively around blood vessels in adults [17]. We utilized this specificity to query the role of RhoJ in fibronectin remodelling around blood vessels in vivo. Using a RhoJ knockout mouse model, we demonstrated that the lack of RhoJ causes increased fibronectin deposition around the developing retinal vessels. This substantiates our observations that RhoJ is a negative regulator of fibrillogenesis. In RhoJ null mice, the vessels have less stability and increased permeability [18]. Our work provides mechanistic insights into this phenotype, suggesting that the negative regulation of fibronectin deposition by RhoJ allows vessels to mature at the end of angiogenesis. Lack of RhoJ could cause persistence of the provisional fibronectin matrix leading to vessel instability. We had previously shown that RhoJ traffics podocalyxin to the early apical surface of endothelial cells thereby aiding in lumen formation [19]. Together, our results suggest that RhoJ inactivation during sprouting and reactivation during lumenisation is necessary for normal angiogenesis. Interestingly, biallelic RhoJ overexpression in the endothelial cells of mice, mimic the phenotype of Cdc42 KO mice, showing severe defects in angiogenesis and embryonic lethality [20]. Our work suggests that this could be due to the overexpression of Rhoj out-competing endogenous Cdc42 function.

A stage-specific role for RhoJ and Cdc42 in the multistep angiogenesis cascade is indicated by the ability of the different growth factors to switch between the two Rho GTPases. For example, the proangiogenic growth factor VEGF is known to activate Cdc42 and inactivate RhoJ [21] while a repulsive cue, Sema3E is known to inactivate Cdc42 while activating RhoJ [20]. We should highlight here that Kaur et al. have reported activation of RhoJ in response to VEGF stimulation over longer time periods [22]. The regulation of RhoJ is likely context dependent as evidenced by a recent study that demonstrates that RhoJ can integrate both the VEGF dependent attractive and Sema3E dependent repulsive cues in endothelial cells [11]. We have not been able to reliably measure RhoJ activation due to issues with the quality of available RhoJ antibodies. It will be important to resolve this apparent discrepancy in the kinetics of VEGF driven RhoJ activation using a more robust activation assay.

A recent study by Prof. Uemura’s group contextualizes the role of RhoJ in endothelial migration during developmental and pathological angiogenesis. RhoJ integrates VEGF and Sema3E dependent signals by altering VEGFR2 receptor binding partners, downstream signalling and receptor fate [11]. The ratio of the attractive (VEGF) to repulsive (Sema3E) cues in the microenvironment dictates RhoJ activation. Consequently, in VEGF high contexts, RhoJ drives VEGFR2-PlexinD1-Nrp1 complex formation, which causes VEGFR2 Y1214 phosphorylation promoting forward migration. On the other hand, Sema3E induces a VEGFR2-PlexinD1 complex devoid of Nrp1, again in a RhoJ dependent manner, that signals through p38 MAPK to promote reverse migration [11]. Significantly, in a context where the VEGF dependent signals predominate in vivo, as seen in the oxygen-induced retinopathy (OIR) mouse model, RhoJ deletion in ECs reduced neovascular tuft formation by 80%. This shows the in vivo requirement of the VEGF-RhoJ axis for forward migration and angiogenesis. This also suggests that RhoJ could be an effective target, under VEGF high contexts, to inhibit aberrant vascularization [11]. Indeed, our work provides the diametrically opposite context involving an intricate balance of attractive and repulsive cues during developmental angiogenesis. Retinal ECs are exposed to VEGF secreted by astrocytes in the same horizontal plane and Sema3E from the underlying neurons. Under these conditions, RhoJ deletion leads to increased fibronectin fibrillogenesis at the angiogenic front [4]. This could be due to the inability of Sema3E to induce actin depolymerization and a reduction in cellular contractility in the absence of RhoJ, that would have acted as a check on the bundling of fibronectin into fibrils [23].

Our study indicates that the functional antagonism between the paralogs RhoJ and Cdc42 is due to competition for shared effectors. Such antagonism is evident in other paralogs within the Rho GTPase family. For example, Rnd proteins paralogous to RhoA-C antagonize RhoA dependent processes [24]. Rnd proteins are known to bind to the RhoA GAP – p190RhoGAP (albeit at a position distinct from that of RhoA), causing an increased GAP activity of this protein towards RhoA [25]. Another example is seen in the antagonism between RhoC and RhoD. RhoD recruits PAK6 to inhibit RhoC dependent cell contractility [26]. There are also reports where closely related proteins bind in a conformation sensitive manner to the same substrate leading to functional antagonism. For example, β arrestins1 and 2, have different affinities for the ligand-bound and unbound conformations of IGF1R, showing antagonistic effects on receptor fate and biological outcomes [27]. It is tempting to speculate that RhoJ, through specific adaptor proteins, might be directly recruited to the active integrin tails to prevent internalization. Both conformers of integrins have very different trafficking repertoires within cells [28]. The ligand-bound active integrins are likely to travel to perinuclear compartments of low pH to facilitate ligand-receptor separation while the inactive conformer is likely to undertake short loop recycling [28]. The presence of RhoJ in large perinuclear vesicles that lack Cdc42 suggests that differential conformation-specific recruitment to and handling of integrins could be key to the functional antagonism between the two Rho GTPases. Indeed, it would be important to delineate the key effector(s) downstream of RhoJ that allows for the regulation of conformation-specific α5β1 internalization. As PAK3 failed to change the initial rate of internalization of the active receptor [4], we would like to speculate that the formins, particularly FMNL3, might play a role. FMNL2 regulates β1 integrin internalization [29] but is reported to be absent in endothelial cells [30]. Therefore, the closely related protein FMNL3, already known to function downstream of RhoJ during lumenisation [19] and downstream of Cdc42 at the Golgi during anterograde trafficking [31] is a likely shared effector that could regulate RhoJ-dependent regulation of active α5β1 trafficking [32].

The functional antagonism between RhoJ and Cdc42 coordinates angiogenesis, suggesting that the retention of the duplicated RhoJ gene, almost exclusively in the cells of the vasculature, serves to dampen the ancestral Cdc42 gene activity during fibronectin fibrillogenesis. The appearance of fibronectin as an ECM protein specific to the chordate lineage [33], and its critical roles in angiogenesis [34] may have fuelled the need to fine-tune its deposition and remodelling. Through the diversification of the roles of the duplicated Cdc42 gene, RhoJ might have ended up taking a key regulatory role in trafficking the fibronectin-bound active integrin α5β1 receptor and fibronectin remodelling.

Endothelial fibronectin deposition in the areas of turbulent blood flow, is the initial trigger for atherogenic inflammation [35]. Subendothelial fibronectin deposition in mature vessels plays a causal role in atherosclerosis [36,37]. Our results indicate that RhoJ might play a critical role in maintaining vessel health by limiting fibrillogenesis in quiescent vessels. We need to perform experiments to directly verify if RhoJ activation could be atheroprotective. In conclusion, our study identifies RhoJ as a novel regulator of conformation-specific integrin trafficking. RhoJ is one of the few negative regulators of fibronectin remodelling known to us, and it serves to counterbalance Cdc42 by competing for shared effectors. The RhoJ-Cdc42 antagonism serves as an example of a unique way in which duplicated genes can acquire new functions through the course of evolution.
